# Prediction of cardiovascular disease risk based on major contributing features

**DOI:** 10.1038/s41598-023-31870-8

**Published:** 2023-03-23

**Authors:** Mengxiao Peng, Fan Hou, Zhixiang Cheng, Tongtong Shen, Kaixian Liu, Cai Zhao, Wen Zheng

**Affiliations:** 1 Institute of Public-Safety and Big Data, College of Data Science, Taiyuan University of Technology, University Street, Yuci District, Jinzhong, 030600 China; 2 Center for Big Data Research in Health, Changzhi Medical College, East Jiefang Street, Changzhi, 046000 China

**Keywords:** Diseases, Medical research, Risk factors

## Abstract

The risk of cardiovascular disease (CVD) is a serious health threat to human society worldwide. The use of machine learning methods to predict the risk of CVD is of great relevance to identify high-risk patients and take timely interventions. In this study, we propose the XGBH machine learning model, which is a CVD risk prediction model based on key contributing features. In this paper, the generalisation of the model was enhanced by adding retrospective data of 14,832 Chinese Shanxi CVD patients to the kaggle dataset. The XGBH risk prediction model proposed in this paper was validated to be highly accurate (AUC = 0.81) compared to the baseline risk score (AUC = 0.65), and the accuracy of the model for CVD risk prediction was improved with the inclusion of the conventional biometric BMI variable. To increase the clinical application of the model, a simpler diagnostic model was designed in this paper, which requires only three characteristics from the patient (age, value of systolic blood pressure and whether cholesterol is normal or not) to enable early intervention in the treatment of high-risk patients with a slight reduction in accuracy (AUC = 0.79). Ultimately, a CVD risk score model with few features and high accuracy will be established based on the main contributing features. Of course, further prospective studies, as well as studies with other populations, are needed to assess the actual clinical effectiveness of the XGBH risk prediction model.

## Introduction

Cardiovascular diseases(CVD) are a group of heart and vascular diseases, namely coronary, cerebrovascular, and rheumatic heart. It has been reported that more than 17.9 million patients die each year worldwide due to heart and vascular diseases^1^. According to the Global Burden of Disease Report 2019^2^, the number of CVD prevalence is steadily increasing, reaching 523 million in 2019, with up to 18.6 million deaths, accounting for one-third of the total deaths^3^. Domestic and international experience shows that early detection and effective intervention management of high-risk populations is a clear technical route and cost-effective prevention and control program that can extend national life expectancy, improve national health and quality of life, and reduce the burden of disease. It has been found that accurate prediction of cardiovascular disease (CVD) requires a variety of information, including not only patient history, but also genomic data, symptoms, lifestyle and risk factors^4^. Therefore, we need to investigate the relationship between risk factors and disease, and use data analysis as a theoretical support to find the intrinsic patterns to achieve accurate prediction of disease occurrence.

In the face of increasing mortality from cardiovascular disease, many institutions have conducted prospective studies on CVD. Typical examples of existing CVD risk prediction models are the PCE cardiovascular risk assessment formula recommended by the American Heart Association/American Heart Association (ACC/AHA)^5^, the European Systematic Coronary Risk Assessment Study^6^, the QRISK cardiovascular risk assessment model published by the QResearch database based disease risk study in the United Kingdom^7,8^, and the Gu The China-PAR model constructed by a research group led by Professor Dongfeng Gu using data from InterASIA (International Cooperative Study of Cardiovascular Diseases in Asia) and China MUCA (1998) (Multicenter Cooperative Study of Cardiovascular Epidemiology in China)^9^, Fátima Sánchez-Cabo PhD et al. based on standardized tests (blood tests and questionnaires ) of a few minimally invasive, routine, quantitative variables of the EN-PESA model^10^, among others. These institutions have introduced risk assessment tools for a variety of diseases such as cardiovascular disease, coronary heart disease, stroke, and heart failure. However, the existing CVD risk assessment models have an implicit assumption that each risk factor is linearly related to the probability of CVD prevalence^11^, which may oversimplify this relationship because it includes a large number of risk factors with nonlinear interactions. Due to their restrictive modeling assumptions and limited number of predictors, these models all exhibit strong geographic and population specificity, and existing algorithms usually do not predict CVD risk correctly^12^, especially for certain subgroups^13^.

In recent years, with high-performance computers, machine learning (ML) has made significant progress in healthcare and medical research^14^. Machine learning models can establish complex nonlinear relationships between risk factors and diseases by minimizing the error between predicted and true outcomes^15,16^. In the field of cardiovascular disease prediction, more typical are Mezzatesta et al.^17^, who used nonlinear SVC for CVD risk prediction in patients in the United States and Italy. Unnikrishnan et al.^18^ who developed SVM-based risk assessment models for predicting the sensitivity and specificity of CVD using eight metrics, and Weng et al.^19^ used four machine learning algorithms including random forest, logistic regression, gradient augmentation machine, and neural network to compare with the established American College of Cardiology guidelines. Although the accuracy of the above models is high, they can impose more burden on patients in clinical settings due to the large number of selected metrics. Therefore, the aim of this paper is to establish a CVD risk score model with fewer features and high accuracy.

In general, the contributions of this paper are described as follows:

- The XGBH model proposed in this paper can save memory space and show better predictive performance by introducing a histogram algorithm. At the same time,the data of 14,832 Chinese cardiovascular patients are introduced to expand the dataset.Finally,the XGBH model is compared with four previous machine learning models to demonstrate the superiority of the model.
- In this paper, by ranking the importance of features on the dataset, a CVD risk prediction model with few features and high accuracy is developed, and the practical value of the four models is evaluated by using decision curves, and finally only three features are needed to make a more accurate risk assessment of CVD.
- Finally, this paper develops a nomogram of cardiovascular disease risk scores. The risk of cardiovascular disease is calculated based on the three screened characteristics, thus enabling the prediction of the probability of a patient’s disease.

In the Results section we describe the baseline features of the dataset in this paper, showing the performance of the proposed model and the evaluation of the model after feature filtering. In the Methods section the source of the dataset and details of the XGBH model are described, as well as a description of the feature filtering methods used in the paper.

## Results

### Characteristics of the study population

The baseline characteristics of the study population are shown in Table 1. Overall, the mean (SD) age of the subjects at baseline was 53.34 (6.77) years, of which 24,470 (35%) were male, 52,385 (74.8%) had normal cholesterol, 59,479 (85%) had normal glucagon, 6169 (8.8%) were smokers, 3764 (5.4%) were alcoholics, and 56,261 (80.4%) had an exercise habit.

**Table 1:** Baseline characteristics of patients.

Variables	Overall	Normal	CVD	*P*-value
	n = 70,000	n = 35,021	n = 34,979	
Age	53 (7)	52 (7)	55 (6)	< 0.001
Gender = male (%)	24470 (35.0)	12107 (34.6)	12363 (35.3)	0.033
Height	165 [159,170]	165 [159,170]	165 [159,170]	0.001
Weight	74.21 (14.40)	71.59 (13.31)	76.82 (14.96)	< 0.001
Ap_hi	120 [120,140]	120 [110,120]	130 [120,140]	< 0.001
Ap_lo	80 [80,90]	80 [70,80]	80 [80,90]	< 0.001
Cholesterol (%)				< 0.001
1	52385 (74.8)	29330 (83.7)	23055 (65.9)	
2	9549 (13.6)	3799 (10.8)	5750 ( (16.4)	
3	8066 (11.5)	1892 (5.4)	6174 (17.7)	
Gluc (%)				< 0.001
1	59479 (85.0)	30894 (88.2)	28585 (81.7)	
2	5190 (7.4)	2112 (6.0)	3078 (8.8)	
3	5331 (7.6)	2015 (5.8)	3316 (9.5)	
Smoke = 1 (%)	6169 (8.8)	3240 (9.3)	2929 (8.4)	< 0.001
Alco = 1 (%)	3764 (5.4)	1941 (5.5)	1823 (5.2)	0.055
Active = 1 (%)	56261 (80.4)	28643 (81.8)	27618 (79.0)	< 0.001

Quantitative variables that obey the normal distribution are described by mean ± standard deviation, and quantitative variables that do not obey the normal distribution are described by median ± interquartile range. The categorical variables are described as quantity and proportion. Statistical differences were determined using the t-test with Welch correction or the Mann-Whitney U test, the Wilcoxon signed-rank test, or the Kruskal-Wallis test. The analysis was performed using RStudio version 7.2 (RStudio, New York, USA).

### XGBH model validation

**Figure 1:**
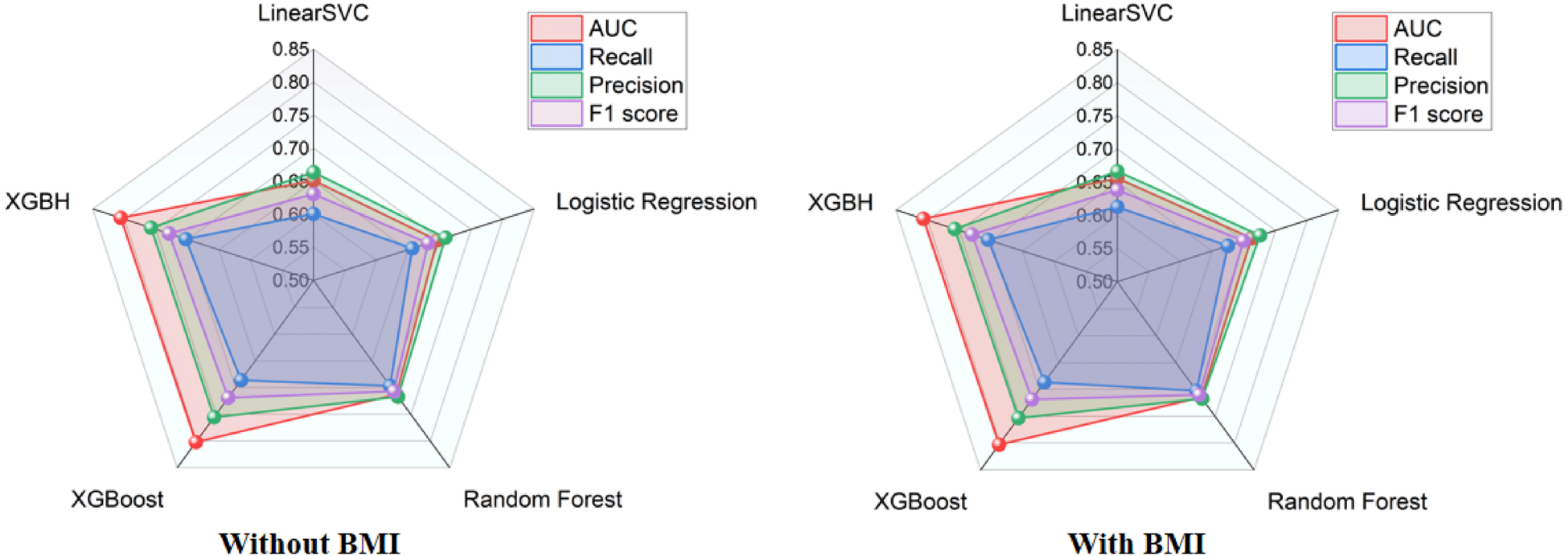
Performance of all prediction models under various feature.

In training the model we use 80% of the kaggle competition dataset and 80% of the Shanxi Baiqiuen Hospital dataset as the training set and the rest of the data as the test set to cross-validate the performance of the model.In this paper, the XGBH model is compared with four machine learning models: logistic regression, linear classification support vector machine, random forest and eXtreme Gradient Boosting (XGBoost), and the classification model is adjusted according to the parameters of the classification algorithm. Table 2 provides the quantitative evaluation results of five machine learning models, logistic regression, linear classification support vector machine, random forest, XGBoost, and XGBH, in terms of AUC, recall, precision, and F1 score.First, as shown in Table 2 with Fig. 1, the XGBH prediction model outperformed the other four baseline models in terms of AUC, recall, precision, and F1 score, regardless of whether the BMI feature was included.In the test group without BMI, the AUC and precision of the XGBH model reached 0.8059 and 0.7578, respectively, indicating that the XGBH model has higher accuracy in predicting CVD risk.We then tried to add a new feature BMI to the prediction model, as shown in Table 2. After adding the BMI feature, there is a small decrease in accuracy, but the rest of the metrics have increased, showing higher predictive power than before. The running time also improved, with XGBoost running time of 4.263 s and XGBH running time of 3.742 s.

**Table 2:** Performance of all prediction models under various feature.

Dataset	Model	AUC	Recall	Precision	F1 score
Without BMI	LinearSVC	0.6511 $-$ (0.6432–0.6589)	0.6006	0.6636	0.6306
	LogisticRegression	0.6965 ± (0.6889–0.7041)	0.6566	0.7094	0.6820
	Random Forest	0.7129 ± (0.7055–0.72044)	0.6967	0.7170	0.7067
	XGBoost	0.8018 ± (0.7945–0.8090)	0.6860	0.7552	0.7190
	XGBH	**0.8059** ± (0.7987–0.8131)	**0.7027**	**0.7578**	**0.7293**
With BMI	LinearSVC	0.6559 ± (0.6480–0.6637)	0.6126	0.6662	0.6383
	LogisticRegression	0.7123 ± (0.7049–0.7198)	0.6749	0.7256	0.6994
	Random Forest	0.7147 ± (0.7072–0.7222)	0.7025	0.7170	0.7097
	XGBoost	0.8027 ± (0.7955–0.8099)	0.6866	0.7532	0.7184
	XGBH	${\textbf {0.8069}}$ ± (0.7997–0.8140)	${\textbf {0.7043}}$	${\textbf {0.7572}}$	${\textbf {0.7298}}$

Values for AUC denoete the mean ± confidence interval (CI). AUC, the area under a receiver operating characteristic cure; $Precesion\,=\,TP/(TP+FP)$, $Recall\,=\,TP/ (TP + FN)$ where TP stands for true positive, TN for ture negative, FP for false positive, and FN for false negative; $F1score\,=\,2(precision*recall)/(precision+recal)$.

### Feature screening and model evaluation

In this paper, we use the PermutationImportance method to analyze the feature importance, which is the contribution of each feature to the prediction, of four models, LogisticRegression, Random Forest, XGBoost, and XGBH, respectively. This is done by randomly arranging the values of a feature column in the dataset to obtain unordered feature values to train the model. Feature importance is identified by observing the degree of influence of feature values on model performance. The feature importance of the target variable is calculated using the PermutationImportance method, and then the feature importance weights are ranked. The descending order indicates the importance weight of each feature, and the feature weight table is drawn as shown in Table 3, from which it can be seen that the models with higher prediction accuracy have closer feature importance ranking. Among them, the two models with the best prediction accuracy, XGBoost, and XGBH, have the same top five important features, namely, systolic blood pressure (Ap_hi), cholesterol (Chol), age, diastolic blood pressure (Ap_lo), and body mass index (BMI). Also, systolic blood pressure (Ap_hi) showed the highest feature weight among all predicted results, indicating that it is the most predictive feature.

**Table 3:** Feature importance of each prediction.

Model	LogisticRegression	Random Forest	XGBoost	XGBH
1	Ap_hi (0.1383 ± 0.0070)	Ap_hi (0.1326 ± 0.0028)	Ap_hi (0.13750 ± .0065)	Ap_hi (0.1406 ± 0.0047)
2	Weght (0.1218 ± 0.0056)	Chol (0.0302 ± 0.0052)	Chol (0.0321 ± 0.0050)	Chol (0.0358 ± 0.0034)
3	BMI (0.0473 ± 0.0030)	Age (0.0239 ± 0.0058)	Age (0.0268 ± 0.0030)	Age (0.0276 ± 0.0043)
4	Height (0.0434 ± 0.0050)	Active (0.0024 ± 0.0016)	Ap_lo (0.0059 ± 0.0007)	Ap_lo (0.0063 ± 0.0007)
5	Age (0.0319 ± 0.0079)	Ap_lo (0.0023 ± 0.0026)	BMI (0.0045 ± 0.0026)	BMI (0.0036 ± 0.0023)
6	Chol (0.0012 ± 0.0008)	Smoke (0.0008 ± 0.0017)	Active (0.0020 ± 0.0011)	Active (0.0034 ± 0.0017)
7	Smoke (0 ± 0.0000)	Gender (0.0002 ± 0.0043)	Height (0.0018 ± 0.0015)	Gender (0.0016 ± 0.0010)
8	Alco ($-$ 0.0000 ± 0.0001)	Alco ($-$ 0.0000 ± 0.0008)	Gender (0.0014 ± 0.0009)	Gluc (0.0015 ± 0.0011)
9	Active ($-$ 0.0001 ± 0.0004)	Gluc ($-$ 0.0012 ± 0.0014)	Smoke (0.0012 ± 0.0009)	Smoke (0.0012 ± 0.0005)
10	Gluc ($-$ 0.0001 ± 0.0003)	BMI ($-$ 0.0092 ± 0.0022)	Weight (0.0010 ± 0.0024)	Weight (0.0010 ± 0.0007)
11	Gender ($-$ 0.0002 ± 0.0003)	Height ($-$ 0.0100 ± 0.0049)	Aclo (0.0003 ± 0.0003)	Height (0.0010 ± 0.0012)
12	Ap_lo ($-$ 0.0003 ± 0.0006)	Weight ($-$ 0.0125 ± 0.0023)	Gluc (0.0002 ± 0.0005)	Aclo (0.0004 ± 0.0006)

The value of weight plus or minus represents the half of 95% confidence interval length. The larger the feature weight, the greater the feature predictability, and the negative weight represents an inhibitory effect on prediction.

**Figure 2:**
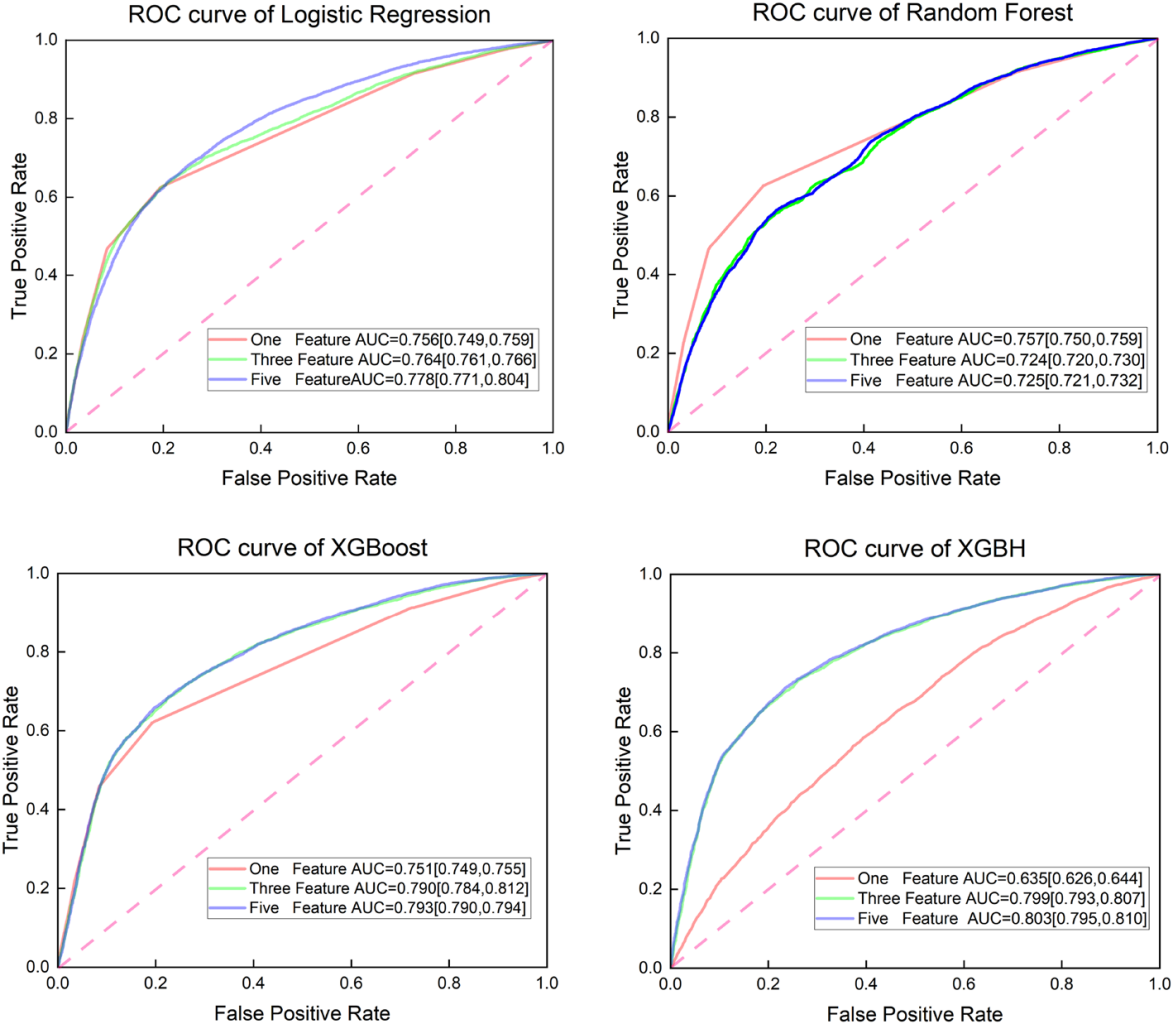
ROC curve, compare the AUC of the four models with different characteristics.

The previous feature importance analysis drove us to build a prediction model based on a small number of important features instead of a model based on all features. According to the order of feature weights of the XGBH model in the Table 3, the presence or absence of cardiovascular disease is the target feature, and four model models are used to make predictions based on different numbers of features and plot the ROC curve, as shown in Fig. 2. As can be seen from the Fig. 2, When using the top 5 features (ap_hi, chol, age, ap_lo, BMI) for prediction, the XGBH model works optimally with an AUC of 0.803 (95% CI: 0.795 to 0.0.810), then using the top 3 (ap_hi, chol, age) features in terms of influence for prediction, the XGBH model predicts still had an optimal AUC of 0.799 (95% CI: 0.793 to 0.807). The results show that when the XGBH model uses the top 5 features for prediction, it can achieve the best results among the four models, and when reducing from five features to three features, the AUC only decreases from 0.803 to 0.7999, indicating that ap_lo, BMI provides less contribution to the increase of model accuracy. All things considered, we used only three questions (1. the value of systolic blood pressure? 2. is cholesterol normal? 3. what is the age?) of the questionnaire will allow for an accurate CVD risk assessment.

**Figure 3:**
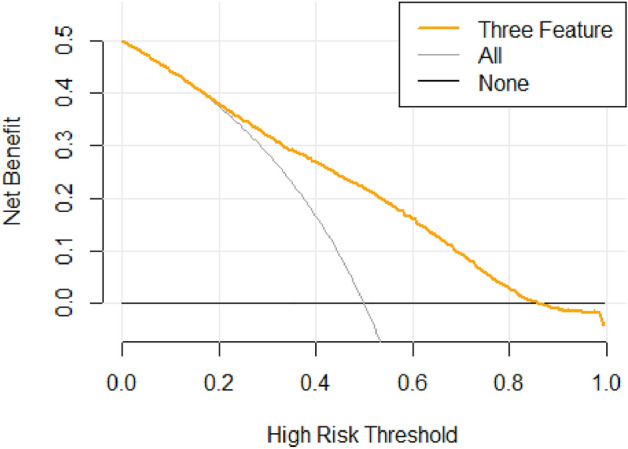
DCA curves for the combined diagnosis of cardiovascular disease with three features.

To consider the clinical benefits of XGBH and compare the application effects of different features in diagnosing cardiovascular diseases, this paper uses Net Benefit as the vertical coordinate and High-Risk Threshold as the horizontal coordinate and draws the DCA curves of 1 feature and 3 features combined to diagnose cardiovascular diseases, as shown in the Fig. 3. The value of the High-Risk Threshold is set to (0, 1); None and All represent two extreme cases, None means that all people do not suffer from cardiovascular disease, and Net Benefit is 0 regardless of the value of the High-Risk Threshold; All means that all people suffer from cardiovascular disease, and Net Benefit changes with the High-Risk Threshold. From the figure, we can see that the Net Benefit is greater than 0 when the High-Risk Threshold is 0.15–0.85, which is clinically significant. And the smaller the value of the High-Risk Threshold between 0.15 and 0.85, the higher the Net Benefit, the greater the clinical significance.

**Figure 4:**
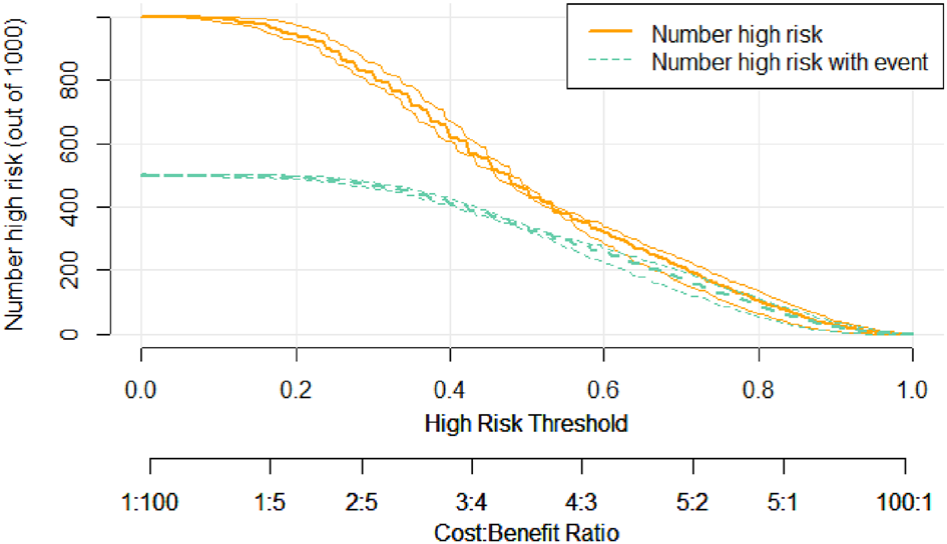
Clinical impact curves.

To further show the diagnostic value of the combination of the three characteristics for the diagnosis of cardiovascular disease, we assumed that there were 1000 patients to get the predicted number of people suffering from cardiovascular disease using the XGBH method and compared with the actual number to get the risk stratification. We plotted the CIC curve, and from the Fig. 4 we can see that the predicted and actual values are closer when the High Risk Threshold is greater than 0.4. the predicted cardiovascular disease using the XGBH method is generally consistent with the actual.

### Scorecard model

**Figure 5:**
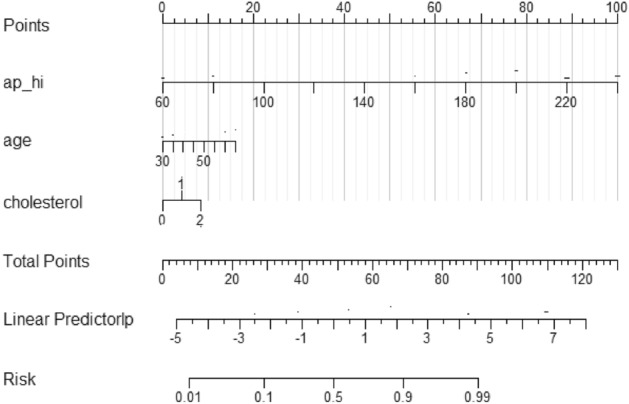
Representation of the XGBH static nomogram.

Although feature importance assessment of machine learning models can build a simple model using a small number of high importance features, the model can only determine if there is a patient with cardiovascular disease, and the risk of disease is not accurately predicted. Based on the nomograms, the score of each predictor can be obtained, and the sum of the scores of all points is the total score of that patient, and the predicted probability corresponding to the total score is the predicted probability of having cardiovascular disease in that patient. In this paper, the XGBH model was constructed using three features and visualized by a nomogram. The length of the line segment as shown in Fig. 5 reflects the contribution of the factor to the outcome event, and from the nomogram, it can be seen that the features with the highest to lowest impact on cardiovascular disease are: ap_hi, age, and cholesterol. and the higher the ap_hi value, the older the age, the higher the risk of cardiovascular disease.

**Figure 6:**
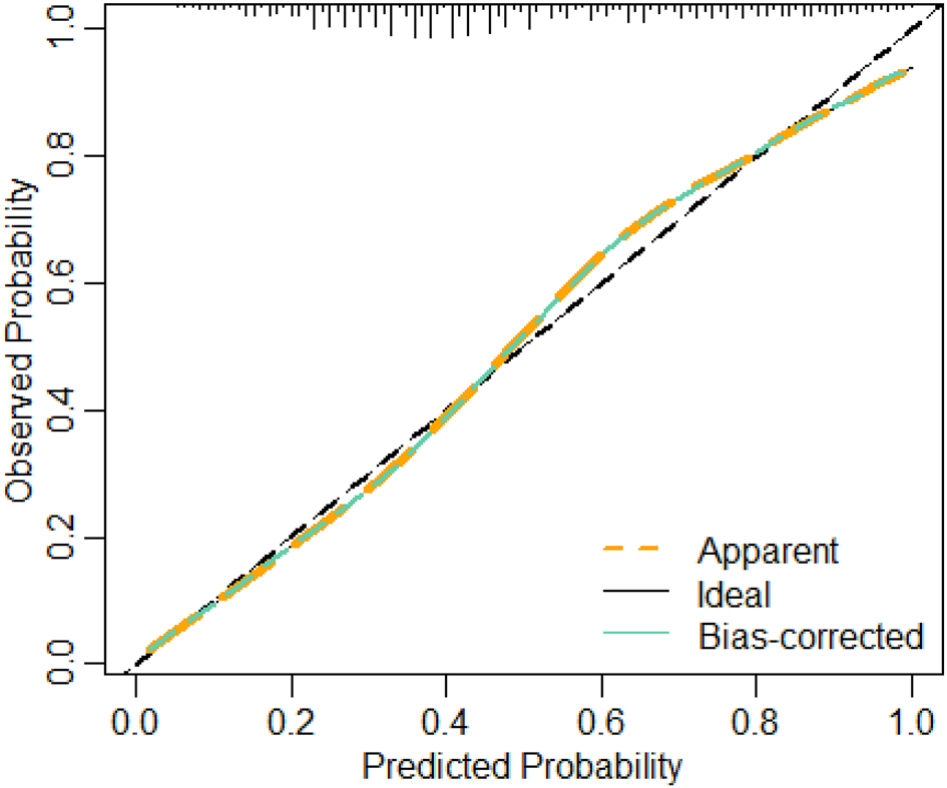
Calibration curve for XGBH.

We plotted calibration curves for evaluating the effect of the nomograms. As shown in the Fig. 6, the Y-axis is the actual probability of developing cardiovascular disease, the X-axis is the probability of developing cardiovascular disease predicted by the model, the diagonal dashed line (Ideal line) indicates the prediction of the ideal model, and the light blue solid line indicates the performance of the nomograms, where the closer to the diagonal dashed line indicates the better prediction performance. The Bais-corrected line represents the performance of the XGBH model trained by repeated self-sampling, which corrects the overfitting situation. When the predicted probability is less than 0.46, the predicted value risk is greater than the actual risk; when it is greater than 0.46 and less than 0.8, it indicates that the predicted value risk is less than the actual risk; when the predicted probability is greater than 0.8, the predicted value risk is greater than the actual risk. Overall, both the calibration curve and the clinical decision curve indicate that the XGBH model built using the three characteristics has high clinical application in predicting the occurrence of cardiovascular disease.

## Conclusion

In summary, this study shows that the XGBH model proposed in this paper requires only three characteristics (1. systolic blood pressure 2. normal cholesterol 3. age) to provide a more accurate risk assessment for CVD. It also outperforms the previous baseline model in terms of model performance and computing time. In conclusion, the method proposed in this paper is more accurate than the existing XGBoost model and is more suitable for predicting CVD risk in a wider range of patients by providing only three indicators of the patient for accurate prediction.

## Discussion

In this work we propose an XGBH model for cardiovascular disease risk prediction based on the main contributing features. The XGBH model in this paper reduces the number of samples and features without loss of accuracy by introducing the histogram idea. The XGBH model compensates for the disadvantages of XGBoost in terms of longer training time and larger memory consumption in use, and shows better prediction performance. Our proposed model achieves the best results in all four evaluation metrics compared to the other four machine learning models logistic regression, linear classification support vector machine, random forest and XGBoost, with AUC,Precision reaching 0.8059 and 0.7578 respectively. The XGBH model predicts the likelihood of CVD occurrence by applying the ML algorithm to treatment data from 70,000 patients in Europe and Asia. The strength of this paper’s study is that with increasing health awareness, most people undergo health screening every 1-2 years, which has contributed to the availability of treatment data for patients. As the prediction model is based on retrospective patient data only, we can use the ML algorithm for simpler and more effective CVD prediction. This approach avoids the additional cost and burden of collecting baseline data compared to traditional CVD prediction models.

Predictive analyses of the risk of disease in cardiovascular patients have been performed in several previous studies, but these were all studies of European patient data, which resulted in models that did not accurately predict in Asian cardiovascular patients due to geographical differences and differences in characteristic information. In contrast, in this study we used a hospital dataset containing data from the real Bethune Hospital in Shanxi, China, including 1913 inpatients with a total of 14,832 medical records, so the results can be more widely applied. Also, the importance of missing values or non-response is not usually assessed when developing conventional cardiovascular disease risk prediction tools^20^. This study suggests that the inclusion of conventional biometric variables such as BMI, in particular, will also improve the accuracy of CVD risk prediction.

We further analysed the features affecting CVD prediction in more depth, and by comparing the feature importance of the four machine learning models we found that the models with higher prediction accuracy were ranked more closely in terms of feature importance. The two models with the best prediction accuracy, XGBoost and XGBH, had the same top five important features, and systolic blood pressure (Ap_hi) showed the greatest feature weighting in all prediction results, indicating that systolic blood pressure was the most predictive feature for CVD.

Several limitations of this study should be mentioned. Firstly, the cardiovascular disease risk prediction study did not include some long text information, which is important for healthcare professionals when diagnosing a patient’s condition, and this could be extracted using techniques such as natural language processing (NLP) to extract useful information from the long text data, which would allow for more accurate prediction models to be constructed and further improve the predictive performance of the models.Secondly, no cholesterol thresholds were disclosed and therefore we were unable to accurately assess the effect of specific cholesterol values on cardiovascular disease. Third, the outliers ap_ho and ap_hi that appeared in the original dataset were not addressed, as attempts to remove the outliers reduced the accuracy of the model. Fourth, the feasibility and acceptability of the new three-question risk assessment model proposed in this paper has not been further investigated in clinical practice.

Moreover, the current study uses a range of machine learning algorithms, which shows an interesting variation in the importance of different risk factors depending on the modelling technique. Decision tree-based models are very similar to each other, and gradient boosters outperform random forests. Neural networks and logistic regression place more emphasis on categorical variables and CVD-related medical conditions, clustering patients with similar characteristics across groups. This may help to further explore various predictive risk factors and the development of new risk prediction methods and algorithms in the future.

In conclusion, we believe that the work in this paper allows for accurate CVD prediction based on a small number of characteristics of European and Asian patients. These results may have many implications for the subsequent treatment of patients. Our predictive model could form the basis of a selective screening process for CVD diagnosis to prevent the development of CVD and its associated adverse health outcomes. Future prospective studies and research with other populations are needed to assess the clinical impact of the model.

## Methods

### Data

The dataset used in this study contains a total of 70,000 samples. They are 14,832 data of Shanxi Bethune Hospital and 55,168 datasets of cardiovascular diseases of kaggle competition, respectively.In this paper, we collected the same data format as the kaggle competition. Each sample has 12 dimensions of features, including four objective features (age, height, weight, gender), four inspection features (Ap_hi,Ap_lo,Chol,Gluc), three subjective features (Smoke,Aclo,Active) and one target feature (Cardio).The dataset contains 35,021 health samples, accounting for 49.97% of the total, and 34,979 disease samples, accounting for 50.03% of the total.The hospital dataset was derived from the data of cardiovascular disease patients stored in Bethune Hospital in Shanxi, China during 2017–2020. The hospital data contains 1913 inpatients, 8179 sick samples and 6653 healthy samples, for a total of 14,832 medical data.

Our hospital dataset is focused on inpatients and includes both structured and unstructured data.Structured data includes laboratory data and basic patient information, such as age, gender and lifestyle. Unstructured text data include patient’s self-report of condition, physician’s questioning and diagnosis results.Since the hospital dataset is entirely Asian, using only this part of the data may result in less accurate prediction of CVD risk in European populations.We therefore added 55,168 kaggle competition cardiovascular disease datasets to the hospital dataset.Objective characteristics include height and weight characteristics, so a new characteristic of BMI was constructed. The detailed characteristic information is shown in Table 4.

The study protocol was approved by the Shanxi Bethune Hospital (Shanxi Academy of Medical Sciences) Medical Ethics Committee (approval number: YXLL-2022-094), and the methods used in this study were conducted in accordance with the approved guidelines. Participants were informed of the objectives and methods of the study, informed consent was obtained from the participants or their guardians by written signature or thumbprint, and they could withdraw from the study at any time without giving any reason.

**Table 4:** Characteristics of the dataset.

Variable type	Feature	Value type	Feature meaning
Objective	Age	Int (years)	Count in years
	Height	Int (cm)	Count in centimeters
	Weight	Float (kg)	Count in kilograms
	BMI	Float (kg/m$^2$)	Body mass index
	Gender	Categorical code	1: women, 2: men
Examination	Ap_hi	Int (mmHg)	Systolic blood pressure
	Ap_lo	Int (mmHg)	Diastolic blood pressure
	Chol	Categorical code	Cholesterol 1: normal, 2: above normal, 3: well above normal
	Gluc	Categorical code	Glucagons 1: normal, 2: above normal, 3: well above normal
Subjective	Smoke	Binary	Whether patient smokes or not
	Aclo	Binary	Alcohol intake
	Active	Binary	Physical activity
Target variable	Cardio	Binary	Presence or absence of cardiovascular disease

BMI = Height / (Weight * Weight).

### XGBH model

XGBH is a fast high-performance gradient enhancement framework, a tree-based learning algorithm^21^. In this paper, by comparing four machine learning models (logistic regression^22^, linear support vector machine^23^, random forest^24^, and XGBoost^25^), the best performing XGBoost model was chosen as the base model. XGBH introduces a histogram algorithm (Histogram)^26^ based on XGBoost, and since in previous studies, XGBoost used a pre sorting method to deal with node splitting, so that the calculated split points are more accurate. However, the training time is long and the memory usage is large in the process of use. The basic idea of the Histogram algorithm is to split the continuous data of each feature into k boxes, i.e. each box is divided into a certain number of data, thus the original continuous data becomes discrete box data. The k discrete boxes are then used to construct a histogram of the k features. As a result, the original need to traverse all the sample points to find the segmentation points becomes a search between boxes, speeding up the rate and reducing memory. And using the histogram to discretize the values of the features will not lose accuracy but will have the effect of regularization, improving the generalization ability of the algorithm. The specific implementation process is as Algorithm 1: 
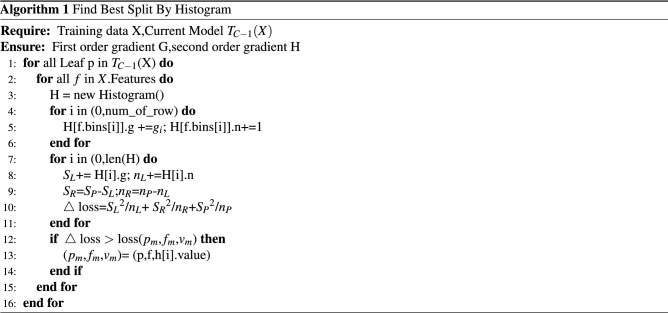


From the algorithm:the histogram optimisation algorithm needs to pre-transform the feature values into bin values before training,make a segmentation function on the values of the features,divide the values of all samples on that feature into a certain segment (bin), and finally discrete the values of the features.

Where H[f.bins[i]].g is the sum of the gradients of the samples in each bin,H[f.bins[i]].n is the number of samples in each bin, $S_L$, $S_R$, $S_P$ represents the gradient sum on the left side of the current bin, the gradient sum on the right side,and the total gradient sum, $n_L$, $n_R$, and $n_P$ represent the number of samples on the left side,the right side and the total number of samples.

### Feature screening and model evaluation

As AI-based predictive models are often black-box models, using them for prediction is often limited by the difficulty of analyzing the causal relationship between risk factors and disease occurrence. Ideally, models are used to provide actionable recommendations for prevention, and the interpretation of what needs to be improved to change an adverse state or identify early risk determines the model’s usefulness. In clinical practice, existing methods require patients to test for more indicators, which increases the burden of use and inconvenience to patients due to the number of indicators required and the complexity of sampling. Interpretable analysis of machine learning models can capture the most influential few features, from which simpler predictive models can be constructed. Therefore, we need to use machine learning models to assess the importance of features in the dataset and to filter them. The basic idea of feature importance assessment^26^ is to calculate the degree of decline in the model performance score by randomly ranking a certain feature, with the more fluctuating values playing a more important role. The specific method is as Algorithm 2.
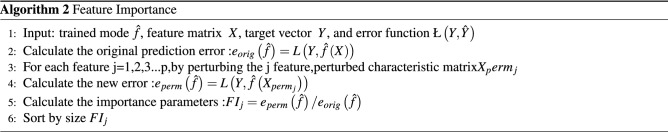


${\hat{Y}}$represents the target vector predicted after training. Finally, the importance of each feature can be obtained through the ordered F$I_j$which is helpful for our subsequent experiments.

This paper’s performance in predicting CVD and datasets is quantitatively evaluated by area under the ROC curve (AUC), recall, precision, and F1score. These metrics will help to identify where the model fails to predict correctly. Some terms help us to calculate these metrics. They include True Positive (TP), which indicates that a positive is correctly identified as a positive. Negative (TN) indicates that a negative is correctly identified as a negative. False Positive (FP) indicates that a negative is incorrectly identified as a positive, and False Negative (FN), See Formula 1, 2, 3 for details.

1 $$\begin{aligned} \text {Recall}&{\text { = }}\text {Sensitivity}{\text { = }}\frac{\text {TP}}{{\text {TP} + \text {FN}}} \end{aligned}$$

2 $$\begin{aligned} \text {Precision}&{\text { = }}\text {PPV}{\text { = }}\frac{\text {TP}}{{\text {TP} + \text {FP}}} \end{aligned}$$

3 $$\begin{aligned} \text {F1-score}&{\text { = }}\frac{2\text {TP}}{{2\text {TP} + \text {FP} + \text {FN}}} \end{aligned}$$

In this paper, using the benign sample group as negative and the malignant sample group as positive, with False Positive Rate as the horizontal coordinate and True Positive Rate as the vertical coordinate, ROC was plotted by SPSS software under four models (LogisticRegression, Random Forest, XGBoost, XGBH) curves, and the AUCs of 1 feature, 3 features, and 5 features for predicting cardiovascular disease were analyzed. In order to consider the clinical benefits of XGBH and compare the application of different kinds of features to diagnose cardiovascular diseases, the DCA curves of 1 feature and 3 features combined to diagnose cardiovascular diseases were plotted to evaluate the practical value of the models.

### Ethical approval and informed consent

Ethics approval was granted by the Shanxi Bethune Hospital (Shanxi Academy of Medical Sciences) Medical Ethics Committee (approval number: YXLL-2022-094). Informed consent was obtained from all individual participants included in the study.

## Supplementary Information

Supplementary Information.

## Data Availability

Some of the data analyzed during the this study are included in the Supplementary Information File and the full data are available upon reasonable request by contacting the corresponding author.
